# Improved Quantification of Cerebral Hemodynamics Using Individualized Time Thresholds for Assessment of Peak Enhancement Parameters Derived from Dynamic Susceptibility Contrast Enhanced Magnetic Resonance Imaging

**DOI:** 10.1371/journal.pone.0114999

**Published:** 2014-12-18

**Authors:** Christian Nasel, Klaudius Kalcher, Roland Boubela, Ewald Moser

**Affiliations:** 1 Department of Radiology, University Hospital Tulln, Karl Landsteiner University of Health Sciences, Tulln, Austria; 2 Center for Medical Physics and Biomedical Engineering, Medical University of Vienna, Vienna, Austria; 3 MR Center of Excellence, Medical University of Vienna, Vienna, Austria; University Medical Center (UMC) Utrecht, Netherlands

## Abstract

**Purpose:**

Assessment of cerebral ischemia often employs dynamic susceptibility contrast enhanced magnetic resonance imaging (DSC-MRI) with evaluation of various peak enhancement time parameters. All of these parameters use a single time threshold to judge the maximum tolerable peak enhancement delay that is supposed to reliably differentiate sufficient from critical perfusion. As the validity of this single threshold approach still remains unclear, in this study, (1) the definition of a threshold on an individual patient-basis, nevertheless (2) preserving the comparability of the data, was investigated.

**Methods:**

The histogram of time-to-peak (TTP) values derived from DSC-MRI, the so-called TTP-distribution curve (TDC), was modeled using a double-Gaussian model in 61 patients without severe cerebrovascular disease. Particular model-based z_f_-scores were used to describe the arterial, parenchymal and venous bolus-transit phase as time intervals I_a_,_p,v_. Their durations (delta I_a,p,v_), were then considered as maximum TTP-delays of each phase.

**Results:**

Mean-R^2^ for the model-fit was 0.967. Based on the generic z_f_-scores the proposed bolus transit phases could be differentiated. The I_p_-interval reliably depicted the parenchymal bolus-transit phase with durations of 3.4 s–10.1 s (median = 4.3s), where an increase with age was noted (∼30 ms/year).

**Conclusion:**

Individual threshold-adjustment seems rational since regular bolus-transit durations in brain parenchyma obtained from the TDC overlap considerably with recommended critical TTP-thresholds of 4 s–8 s. The parenchymal transit time derived from the proposed model may be utilized to *individually* correct TTP-thresholds, thereby potentially improving the detection of critical perfusion.

## Introduction

Although promising approaches were published so far, cerebral perfusion measurement using dynamic susceptibility contrast magnetic resonance imaging (DSC-MRI) still lacks a reliable procedure for quantitative measurement of regional cerebral blood flow [1], [2], [3]. Alternatively peak-enhancement parameters judging hemodynamic perfusion aspects, such as time to peak (TTP), standardized time to peak (stdTTP) or time-to-maximum (Tmax), also provided by DSC-MRI are used instead in diagnostic routine and in clinical studies [4], [5], [6], [7], [8].

However, calculations of TTP and Tmax are quite ambiguous, since already altering the model fitted to the voxel concentration-time curves leads to a significantly differing size of the predicted critically hypoperfused volume [9]. Additionally, Tmax critically depends on selecting an appropriate arterial input function (AIF) for deconvolution of the concentration time curves, which most likely is a reason why this parameter appeared more robust than TTP in one study, while other trials could not find any advantage in using Tmax instead of TTP [10], [11]. Another peak enhancement parameter, which only requires critical sampling of the cerebral bolus transit during its first pass, while keeping the contrast level of the sequence acceptably high, is stdTTP [6]. This technique reverts to the voxel peak enhancement time only to enable a global analysis of the contrast distribution in the brain over time, without the need to specify any model-fitting or AIF, and proved useful in acute stroke as well as in stenotic carotid disease [12], [13].

Commonly, using any of the proposed peak enhancement parameters to differentiate between regular and critical perfusion requires the definition of a certain delay time threshold, where voxels showing time values higher than this threshold are considered to be at risk for ischemic injury. Studies trying to define the one ideal generally valid threshold for TTP and Tmax by testing the parameters against positron emission tomography found a threshold range from 4 s–8 s only, which suggests relevant individual variations of the critical perfusion thresholds [14]. Compared to TTP and Tmax, which lack a practical recommendation, how to exactly handle the data during the calculation, stdTTP provides a clear concept of how to calculate and judge regional bolus transit delays. Although valid thresholds for regular and critical perfusion, of 3.5 s and 7 s respectively, were defined for stdTTP, a so called tolerance interval between these extremes had to be postulated, which likewise seems to result from individual variations of the critical brain perfusion threshold [12].

While for TTP and Tmax at least some variations in the assessment of critical perfusion can be traced back to ambiguities in the calculations, no such inconsistencies are possible for calculating stdTTP, where for any given DSC-MRI examination only one distinct solution exists [6], [12]. Thus, evidently, all peak enhancement parameters, independently from their calculation method, are therefore hampered by their rather ambiguous threshold definitions and this may become even more troublesome when only one single threshold is used to reliably demarcate critically perfused tissue in various patients. Consequently, the single threshold concept has to be questioned.

Therefore, we derived individualized thresholds from an unambiguous global cerebral bolus transit analysis. The aims of this study were (1) to compute individualized thresholds by (2) ensuring the comparability of the data across patients, and (3) to explore the physiological range of these thresholds.

## Methods

### Patients

Patients who received DSC-MRI examinations at our institution were selected from our database (n =  app. 5881) using randomly generated study dates (http://www.random.org). Inclusion criteria were: (1.) sufficient image quality of DSC-MRI to calculate perfusion maps, (2.) no sign of focal diffusion restriction in diffusion weighted MRI at a b-value of 1000 s/mm^2^, (3.) no tumor or other defects (e.g., due to infarct or bleeding) with a size >5 mm, (4.) no sign of any other focal disturbance of the blood-brain-barrier or of a vascular malformation, (5.) no cerebrovascular stenotic or occlusive process detectable on MR-angiography (range: aortic arch to the circle of Willis), and (6.) no severe, presumably microangiopathic alterations ( =  grades 2 and 3 according to the Fazekas 4-grades scoring system [15]). Approval of the study, registered under: GS4-EK-4/220-2013, was obtained from the Lower Austrian Ethics Commission. Informed consent of retrospectively acquired data from the database was not required since all data were analyzed anonymously. Prospectively included patients (stroke examples) gave written informed consent prior to participation in this study and were also processed anonymously in the further work up.

### Magnetic Resonance Imaging

All examinations were performed on a clinical 1.5T MR-scanner (Avanto, Siemens Medical Systems-Erlangen, Germany). The standard perfusion protocol (54 patients) included DSC-MRI, diffusion weighted MRI (dual-b SE-EPI: b1 = 0 s/mm^2^; b2 = 1000 s/mm^2^), MR-angiography and conventional MRI including enhanced PD/T2w-IR-imaging [16]. DSC-MRI was performed using a short-TR dynamic contrast enhanced T2*-weighted single shot GRE-EPI-sequence (TR = 689 ms/TE = 17 ms/FOV = 240 mm/slice thickness: 6 mm/image matrix: 164×208 voxels) that allowed acquisition of 81 image-stacks, with 20 slices each, at a scan time of approx. 60 seconds. Additionally, 2 patients (female, 81 a; male, 44a) suffering from acute stroke, who were examined with the standard perfusion protocol, could be assessed with the proposed method in order to gain some preliminary data about potential limitations of individual threshold adaptions. Corresponding diffusion weighted imaging from follow-up examinations >48 h after stroke were compared to the initial infarct prediction of z_f_-score maps obtained in the acute phase. Alternatively to the standard perfusion protocol, a comparable GRE-EPI-sequence (7 patients) with: TR = 1030 ms/TE = 16 ms/FOV = 240 mm/slice thickness: 6 mm/image matrix: 204×204 voxels was employed for DSC-MRI, which enabled acquisition of 50 image-stacks, with 20 slices each, within approx. 52 seconds. The corresponding sampling rates for the global brain bolus passage were 1.45 Hz (TR = 689 ms) and 0.97 Hz (TR = 1030 ms), respectively, for an expected regular minimal arterio-venous bolus-transit time of about 3.5 s [17], [18].

### Modeling of the Global TTP-Distribution

**Figure 1:**
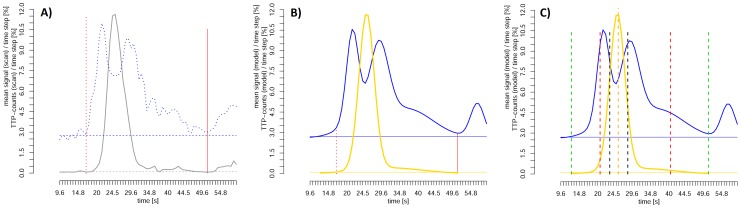
Example of a global perfusion event derived from DSC-MRI. **A**) The time-to-Peak (TTP)-distribution curve (TDC) describes the chronological arrangement of the relative amount of voxels showing their peak enhancement at a certain time step during the examination (grey solid lower line). In parallel the mean signal curve (MSC) (blue dotted upper line) for all voxels with the same TTP-value is displayed in relation to the TDC. Mind the characteristic double peak structure of the MSC that was always arranged around the main TDC-peak. The red vertical lines mark the estimated beginning (dotted) and end (solid) of the wash in and wash out phase, respectively. **B**) After extraction of irrelevant fluctuations in the mean signal curve (blue solid line) the residual components were used to define an arterio-venous transit interval (marked by vertical dotted and solid red lines). Within this interval a double Gaussian model was fitted to the main TDC-peak (gold solid line). **C**) In the last step of the analysis, based on the fitted double Gaussian model z_f_-scores were computed and the time points (marked by vertical lines) for integer scores of z_f_ = −/+3 (green dashed lines), −/+2 (red dashed lines), −/+1 (black dashed lines) and 0 (orange dashed – extended line) were estimated. These z_f_-scores, which were derived from the fitted model, served then to create vascularity maps, which allowed a differentiation between large arteries and veins as well as parenchymal vessels without any a priori defined absolute time thresholds.

Anonymized DSC-MRI examinations were transferred to a PC-work station and stdTTP-maps were computed using in-house developed software (jPerfusionModule; v 2.0.0; available from the corresponding author). Hypoperfusion was ruled out in all cases by stdTTP, which is described in detail elsewhere [6], [12]. The computing kernel of stdTTP provides also a global TTP-distribution curve (TDC) and the correlated mean signal curve (MSC). The TDC describes the chronological arrangement of TTP values of an examination and displays for each time-step of the dynamic scan the relative frequency of voxels showing a peak-enhancement time equal to the respective time-step. The MSC displays the correlated averaged relative signal measured in the voxels from at each time-step of the TDC (Fig. 1A).

Further assessment was performed using the software packages R (version 3.0.1) and SPM8 (Statistical Parameter Mapping 8, UCL-Wellcome Trust Centre for Neuroimaging) [19]. The MSC was smoothed using normal mixture modeling for model-based clustering and was used only to estimate the time points for the arrival of the contrast bolus in the skull and the end of the first pass [20]. The time of the bolus arrival was assumed when the MSC exceeded the pre-contrast base line threshold (mean_BL_+2.5*SD_BL_; SD … standard deviation) for at least 2 s, while the end of its first pass was set at the time point, when the MSC-signal level fell below approx. one third of the MSC-peak signal to a distinguishable local minimum or the end of the scan was reached. Then the TDC was fitted to a double-Gaussian model within this interval using finite mixture modeling that incorporated the expectation-maximization algorithm (Fig. 1B) [21]. The double-Gaussian model was chosen assuming at least to different vessel components with an early fast filling arterial and a late slower filling venous phase. In this model parenchymal voxels were expected to be represented as a mixture of these two major components in the middle of the TDC main-peak. Means, SDs and probability weights provided by the model were used to derive a standard normal distribution based z_f_-score for each TDC time point or TTP-value, respectively, where the probability weights correspond to the proportional probability of the respective fitted single Gaussian curve in the model. The distribution function 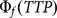 of the normal distribution served to calculate quantiles for each TDC time point according to the model (equation 1):
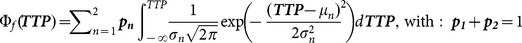
(1)where *TTP* applies to a given time point in the TDC and *µ*, *σ* and *p* are the mean, SD and probability weight of the respective Gaussian sub-function in the model. Since double Gaussian fitting was performed the maximum value for n was 2. Thereafter, standard normal distribution based z_f_-scores were calculated for 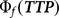 using the R built-in approximation algorithm AS 241 for estimation of quantiles of the normal distribution [22]. Thus a distinct z_f_-score could be assigned to each TTP-value and vascularity maps containing the voxels (v) in the following three time intervals: I_a_ = {v |−2 ≤ z_f_(TTP_v_) <−1}, I_p_ = {v |−1 ≤ z_f_(TTP_v_) ≤ +1}, I_v_ = {v |+1< z_f_(TTP_v_) ≤+2} were generated, where z_f_ means the generic z-score according to the applied model that corresponds to the individual TTP-value measured in a certain voxel v.

### Individual Threshold Calculation and Statistical Analysis

In a second step TTP-values corresponding to z_f_ = −3, −2, −1, 0, +1, +2, +3 were computed iteratively (Fig. 1C) and the interval durations between these limits were calculated as: ΔI_a_ = TTP_(−1)_ −TTP_(−2)_, ΔI_p_ =  TTP_(+1)_ −TTP_(−1)_ and ΔI_v_ = TTP_(+2)_ −TTP_(+1)_. Where, more formally TTP_(zf)_ corresponds to f^−1^(Φ(z_f_)), with f^−1^ being the inverse of the distribution function Φ(z_f_) of the fitted model.

About ∼65% of all voxels of the total intracranial volume were found to represent brain parenchyma in space defining analysis (probability level >0.9) using SPM8. Since the TDC displays parenchymal voxels around the top of the main-peak and, according to the applied model, the TDC interval Ι_p_ with z_f_-limits: −/+1 includes 68.27% of all voxels, the interval Ι_p_ should describe the parenchymal bolus transit best. Consequently its duration ΔΙ_p_ can be interpreted as a measure of the individual regular perfusion threshold, as this should be the maximum delay possible for regularly perfused parenchyma.

**Figure 2:**
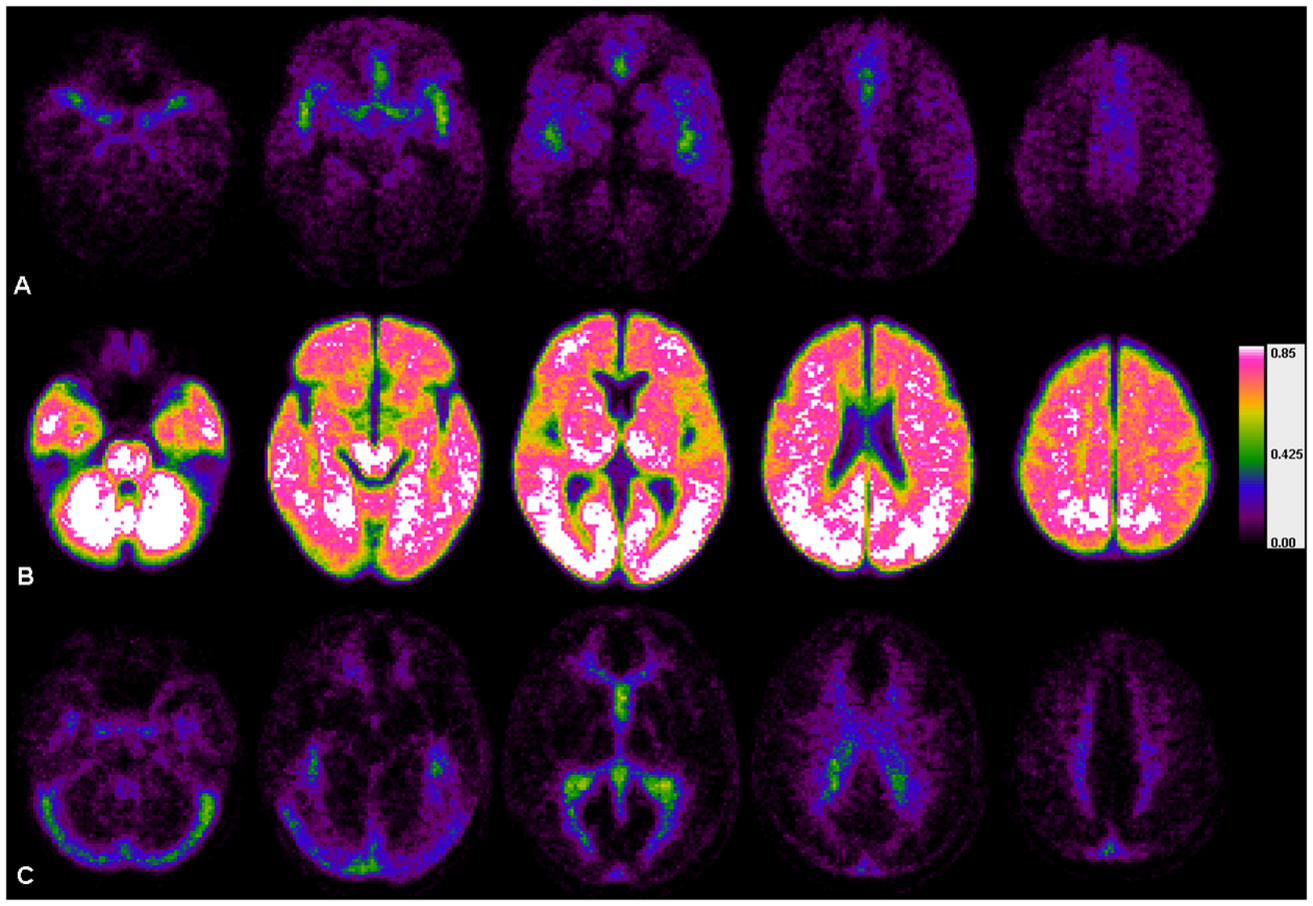
Cumulative vascularity maps (n = 54; TR = 689 ms) of z_f_-intervals. **Row A**: Ι_a_ = {v | −2≤z_f_(TTP_v_) <−1}, **row B**: Ι_p_ = {v |−1≤z_f_(TTP_v_) ≤+1} and **row C**: Ι_v_ = {v |+1 <z_f_(TTP_v_) ≤+2}. Using the proposed z_f_-intervals regions containing mainly arterial vessels (**maps in row A**), regions containing mainly capillary and small cerebral vessels (**maps in row B**), and regions mainly containing veins and sinuses (**maps in row C**), could be easily differentiated. Note, that the interval durations for the different maps were solely derived from the global TTP-distribution, which could be used for individual adjustments of vessel segment specific thresholds. An excellent agreement (shown in green – white colors) between the different exams could be demonstrated, especially for the parenchyma. Anatomical variability between patients, which is expected to be greater concerning the individual shape of the arterial and venous vessel tree, as well as the known technical limitations of echo planar imaging are precluding a 100%-match of the z_f_-score based compartment-classification in the tested samples.

**Figure 3:**
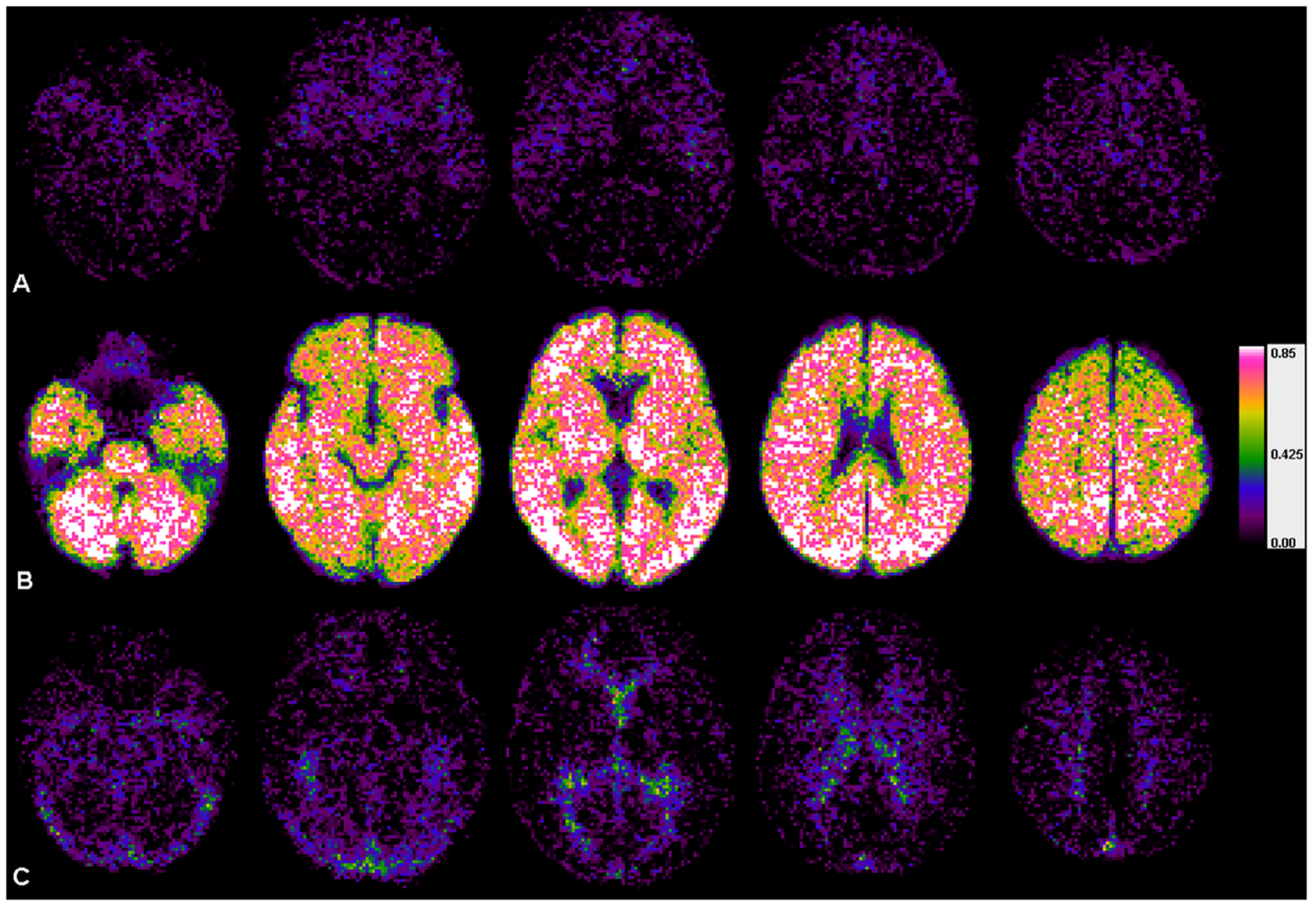
Cumulative vascularity maps (n = 7; TR = 1030 ms) of z_f_-intervals. **row A**: Ι_a_ = {v |−2≤z_f_(TTP_v_) <−1}, **row B**: Ι_p_ = {v |−1≤z_f_(TTP_v_) ≤+1} and **row C**: Ι_v_ = {v |+1 <z_f_(TTP_v_) ≤+2} (same as Fig. 2). Using the proposed z_f_-intervals, independently of the clearly longer TR, regions mainly containing arterial vessels (**maps in row A**), regions containing capillary and small cerebral vessels (**maps in row B**), and regions mainly containing veins and sinuses (**maps in row C**), could be readily distinguished from each other. (Note that due to the smaller number of patients in this group the maps appear less homogeneous and more noisy than those in Fig. 2.)

All individual vascularity maps were then coregistered to the corresponding anatomical MR-images and spatial normalization to MNI-standard space, as provided by SPM8, was performed. Cumulative vascularity maps showing the probability of each voxel to belong to either of the intervals were generated for measurements with short and long TRs (Figs. 2 and 3), to test for the ability of the described method to identify parenchymal perfusion.

Finally, quality of fitting was controlled by calculation of R^2^ between the respective TDC and the associated fitted model curve (*f*TDC) as (equation 2):
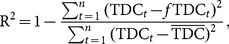
(2)where 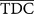 is the mean of the observed data, and TDC_t_ and *f*TDC_t_ are the TDC- and fitted-TDC-values at a certain time point *t*.

Generally, descriptive statistics for interval scaled data, also performed in R-statistics, were computed as median, minimum, maximum as well as 5%- and 95%-percentiles for time based data, otherwise mean, SD or/and confidence intervals are quoted. Group-level correlations were tested using Spearmen rank-correlation tests.

## Results

In total, 61 patients (27 female: 58.0±15.5 years, 34 male: 59.3±13.2 years) were included.

**Figure 4:**
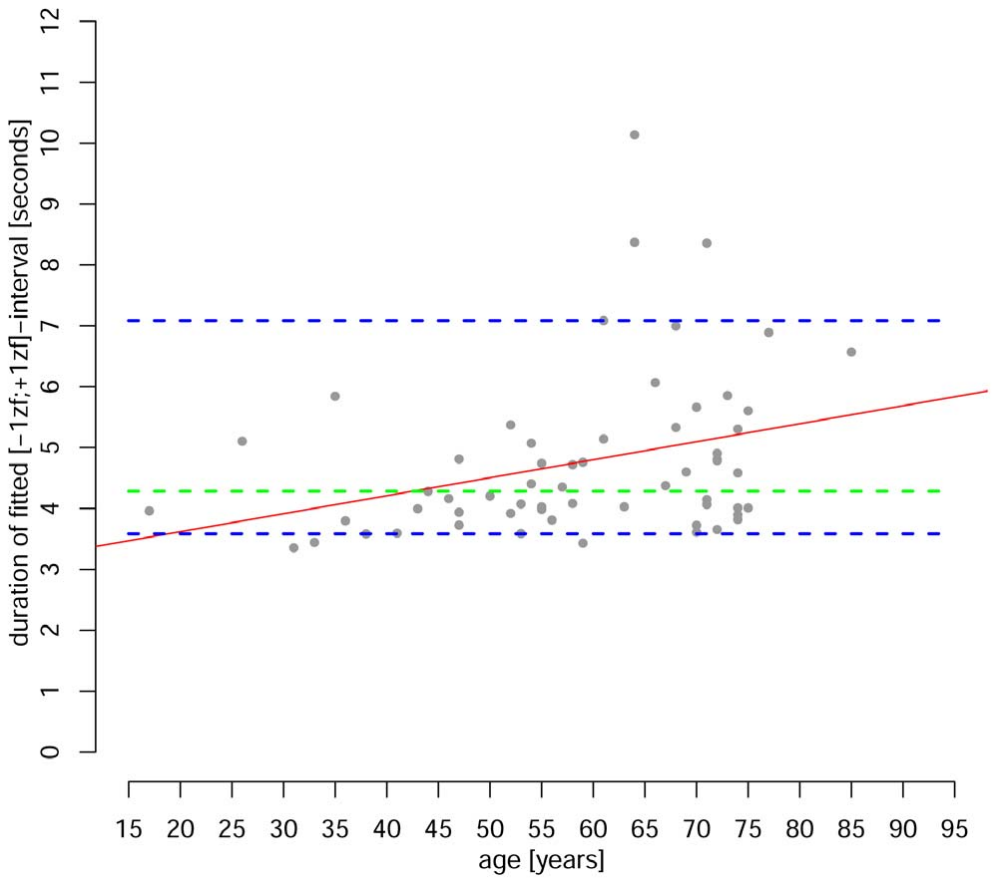
Distribution of durations found for the interval Ι_p_ =  {v |−1≤z_f_(TTP_v_) ≤+1} are depicted in relation to the patient age. An increase of approx. 30 ms per year on average was obtained from the linear regression analysis (red trend line). The dashed horizontal, blue lines mark the 5%- (lower) and 95%- (upper) percentiles of the Ι_p_ - interval, where the median shows at 4.3 s (green dashed horizontal line).

The interval I_p_ showed the parenchymal perfusion phase on the subject- as well as the group-level best. The median duration for ΔI_p_ was 4.3 s (n = 61) with a range between 3.4 s [min] and 10.1 s [max], where the 5- and 95-percentiles were 3.6 s and 7.1 s, respectively. Linear regression analysis (n = 61; model: ΔI_p_∼age; g(x) = 0.03x+3.1; p = 0.012; R^2^_adj_ = 0.086) demonstrated an increase of ΔI_p_ with increasing age (Fig. 4).

Evaluations with respect to TR showed the median of ΔI_p_, when TR = 689 ms was used, at 4.3 s (n = 54) with a range from 3.4 s [min] to 10.1 s [max]. The 5- and 95- percentiles were 3.5 s and 6.9 s, respectively. For TR = 1030 ms (n = 7) the median of ΔI_p_ was: 4.1 s, where the values ranged from 3.8 s [min] to 8.4 s [max].

**Figure 5:**
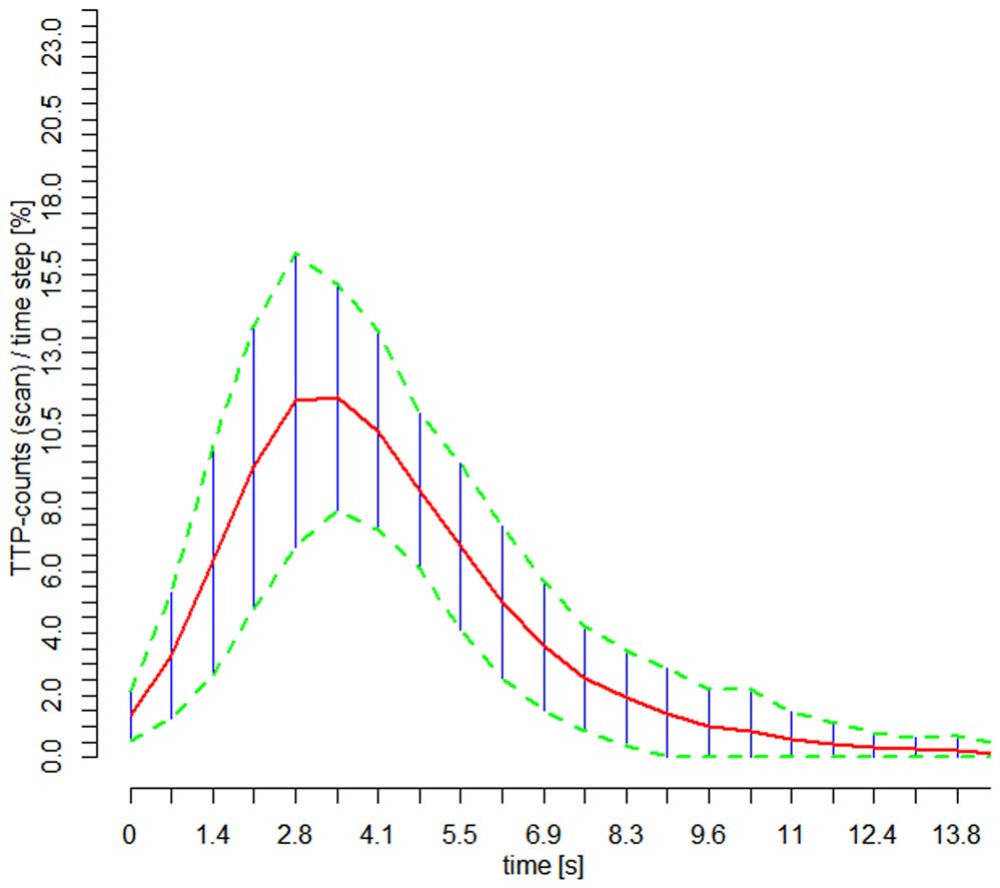
When overlaid within comparable time intervals, the raw time-to-peak distribution curves (TDC) showed considerable variance, but their similarity of the basic structure is evident, which is also reflected by the moderate correlation across patients (ρ = 0.86; Spearman rank correlation; n = 61). The mean curve (red line) and the interval: mean −/+1*SD (green dashed lines) are shown.

The correlation of TDCs before fitting for TR = 689 ms was 0.86 (mean-Spearman's rank correlation coefficient; n = 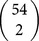), where the median for intersubject correlations was at 0.94 and the 5 and 95- percentiles were 0.43 and 0.99, respectively (Fig. 5). In examinations with TR = 1030 ms, the TDC correlation was 0.75 (mean-Spearman's rank correlation coefficient; n = 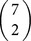). The median was at 0.75 and the 5 and 95- percentiles were 0.40 and 0.96, respectively. The over-all TDC correlation before fitting was 0.85 (mean-Spearman's rank correlation coefficient; n = 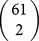), where the median was at 0.92 and the 5 and 95- percentiles were 0.42 and 0.99, respectively.

R^2^ for double-Gaussian fitting was 0.967 (mean) with a confidence interval for mean-R-squared with α = 0.05 and n = 61 of 0.952-0.981.

## Discussion

Modeling the parenchymal cerebral bolus transit in patients without manifest cerebrovascular disease provided evidence that normal bolus transit durations significantly vary and potentially overlap with the postulated range of critical perfusion thresholds for the frequently used peak-enhancement parameters [5], [7], [8]. This agrees to the inability of numerous studies to exactly define one single valid threshold that reliably demarcates critical from regular perfusion in different examinations [1], [2], [3], [9], [10], [11], [14]. Replacing the single threshold concept in favor of an individualized threshold concept should be considered.

Fitting the proposed double-Gaussian model to the TDC enables to switch between model-based generic z_f_-scores and correlated individual TTP-values. Consequently, generic z_f_-scores warrant the comparability of measurements across patients. Furthermore, the reliability of the link between z_f_-scores and individual TTP-values was demonstrated by the excellent correlation between the model and the TDC in all patients (mean-R^2^: 0.967). Therefore z_f_-score-based bolus transit intervals defined in this study always relate to the same physiological periods in the fitted model, but correspond to individually different absolute bolus transit durations. This opens a sensible way to measure durations of individual bolus transit intervals and to correct a threshold according to the given individual lengths, since the bolus transit duration is known to depend on various hemodynamic factors [10], [13], [23], [24], [25], [26]. Moreover, the intended threshold correction in this study is an inherent part of the proposed model, because any threshold defined by a certain z_f_-score automatically translates into case-specific time values and vice versa. We were able to prove the validity of this assumption by generating vascularity maps of intervals I_a_, I_p_ and I_v_, which were defined by generic z_f_-scores only and showed the same bolus transit phases in all patients, despite different absolute durations of these phases in the individual examination. In concordance to simulations prior to the evaluations the interval I_p_ contained ∼68.3% of all voxels in the middle part of the TDC main-peak with a probability >90% to represent cerebral tissue. The interval I_p_ clearly depicted the brain parenchyma independently from the absolute TTP-values, while intervals I_a_ and I_v_ reliably displayed the arterial and venous phase of the bolus-transit in *all* cases (Figs. 2A,B,C and 3 A,B,C).

Under conditions of regular brain perfusion the duration ΔI_p_ of the parenchymal bolus transit interval I_p_, defined by generic z_f_-score limits: −/+1, varied considerably from 3.4 s to 10.1 s, within 90% of all patients exhibiting transit times between 3.6 s and 7.1 s. This remarkably corresponds to the proposed thresholds of 3.5 s and 7.0 s for regular and critical perfusion for the stdTTP-parameter [6], [12], on the one hand, and also matches the common thresholds for TTP and Tmax, ranging from 4 s to 8 s, on the other hand [10], [14], [29], [30]. One study comparing critical perfusion measured with DSC-MRI directly to positron emission tomography measurements in the same patients postulated a threshold range of 3.9 s–6.6 s, with a mean of 5.5 s, for Tmax and a range of 2.8 s–5.8 s, with a mean of 4.2 s, for TTP [10]. All these findings together reveal a variability comparable to the variation found for the bolus transit duration in our study. Furthermore, the inter-subject correlation between DSC-MRI and positron emission tomography in the same study was not significant, while on the subject-level such a correlation was very well found. The latter finding is highly suggestive that probably the lack of a subject-specific normalization that would have corrected the individual variances - which is the proposed individualization of the threshold in our study - was the reason why no correlation on the inter-subject level of the analysis was found in this former study. Considering the high variance of the regular bolus transit duration of about 3.5 s, found in our study, this again emphasizes the need to individually adjust the thresholds and provides further evidence of the limitations of the single threshold concept.

Furthermore, using one single threshold relies on comparing lags of the regional bolus transit to a previously determined maximum delay that safely separates critical from regular perfusion. This assumes a highly homogenous bolus-transit in all examinations, because TTP depends on the arterio-venous transit [23], [27]. In our study the cerebral bolus passage was described by modeling the TDC that reflects the global chronological distribution of TTP-values. Evidently, the chronological order of TTP-values in the course of the TDC is not arbitrary, but determined by the rather rigid concept of a cascaded filling of various vessel segments, which forms the basic structure of the TDC and complies with the arterio-venous bolus transit. Comparably to the proven concept of early and late filling vessel segments in intra-arterial angiography this leads to a tight relation between TTP, vascular anatomy and the arterio-venous bolus transit [28]. Variations of the TDC are most likely caused by numerous, mostly hemodynamically meaningful, factors which modify length and amplitude of the cerebral bolus transit, like cardiac output, age, gender, the extent of global degenerative cerebrovascular disease, but also by methodical aspects, like the chosen injection protocol, etc. [23], [26], [27], [28]. Despite this, the rather similar basic structure of the raw TDC found in all examinations (mean-ρ: 0.86) could primarily result from the same inner circulation structure (ICS) of the brain dictated by the comparable cerebrovascular anatomy in all patients. This similarity is obviously not strong enough to overrule the effect from every factor and so to enable the definition of a single threshold valid for all DSC-MRI examinations, but could explain why single threshold concepts worked at least to a certain extent in the past.

On the other hand, our model conforms to a generic description of the sum of implications from the ICS and other factors on the global bolus transit, which neither requires absolute homogeneity of the data, nor a correction of all factors, since the comparability of the data is accomplished inherently by the model used. For instance, assessing the proposed parenchymal interval I_p_, we found a trend for an increase of ΔI_p_ of 30 ms per year of life (Fig. 4) that confirms earlier reports [31]. Additionally, a certain dependency of absolute TTP-values on TR seems plausible, because higher TRs could introduce a bias due to undersampling of the time-contrast curves. Since fitting certain models to the undersampled curves was also shown to introduce an additional error [9], the prescribed sequence should better be balanced between sufficient contrast and sufficient time-resolution. However, the z_f_-score based interval definitions were not affected by these findings and the various bolus transit phases were reliably identified across patients, while individual variations were well preserved in the absolute TTP-measurements. This seems important, since parameters like Tmax, which attempt to reliably eliminate all spurious run time delays, are also losing hemodynamically relevant information [10].

**Figure 6:**
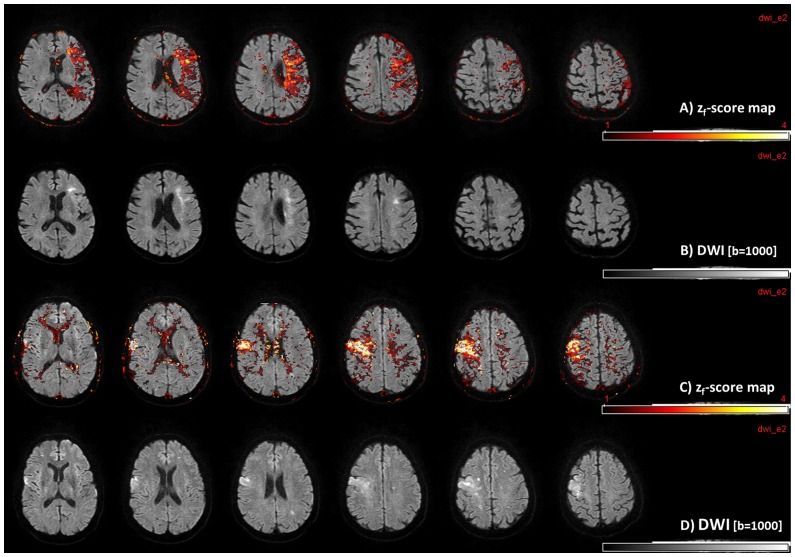
Exemplary use of TDC-modeling. TDC-modeling adapts thresholds automatically, since the same z_f_-score corresponds to individually different absolute thresholds. The TDC interval: [+1< = z_f_< = +4] (overlain with correlated diffusion weighted imaging [DWI]) is shown in **rows A** and **C**. Areas colored in yellow in the TDC-maps display z_f_-scores> = +2 (putative critical perfusion), while red colored regions mark areas with z_f_-scores <+2 (putative tolerable hypoperfusion). In **rows A** and **B** acute cardioembolic stroke is depicted, where only few yellow areas with critical perfusion (**row A**) match well with the DWI-alterations (**row B**). The same behavior was found in the patient shown in **rows C** and **D**, who suffered thromboembolic stroke due to acute occlusion of the internal carotid artery on the right and a high grade stenosis on the left side. Again a good match of the TDC prediction (**row C**) with real ischemic injury in DWI-images (**row D**) on both sides was found. Note that although the same z_f_-scores were used in both cases and no severe overestimations for critically perfused areas occurred, the automatically calculated individual critical thresholds were 9.7 s for case 1 (**rows A & B**) and 5.8 s for case 2 (**rows C & D**).

However, our study has several limitations. With respect to critical sampling of the bolus transit rather short TRs were used. Nevertheless, a serious impact on our investigations is unlikely, as signal levels were found sufficient and no other data quantification step except calculating TTP was performed. Additionally, the shapes of MSC and TDC could change when using a spin echo - instead of a gradient echo EPI technique [17]. Therefore, future applications of our approach will be restricted to a direct assessment of the TDC, as the TDC is expected to remain quite unchanged in different examination techniques. Furthermore, in case of ischemia, double-Gaussian fitting could be hampered by an increasing number of voxels with late peak enhancement. On the other hand, as brain parenchyma represents the largest fraction of voxels in the TDC, double-Gaussian fitting should remain stable in acute stroke. Additionally, voxels with only weak correlation to the proposed model can be easily identified, which, for instance, could be used to define the infarct core in acute ischemia more accurately. To what extent z_f_-score based thresholds, which translate into different absolute time thresholds, will effectively reduce over- or underestimations of impending brain damage in ischemia remains yet to be proven. Nevertheless, first anecdotic data presented here by standard viewing software appears promising to correct spurious overestimations [32] (Fig. 6). At this point further investigations on this topic including a much bigger sample are clearly required.

## Conclusions

TDC-modeling enables unrestrained switching between individual TTP-values and generic z_f_-scores, which maintains comparability across patients, while preserving all hemodynamically relevant individual data. Defining generically comparable, and now individualized, thresholds to assess TTP seems therefore feasible. Investigating individual durations of the parenchymal bolus transit determined by generic z_f_-scores revealed regular transit times significantly overlapping with threshold-ranges postulated for critical perfusion in DSC-MRI using peak enhancement parameters. Thus adapting applied thresholds to the individual parenchymal transit duration seems to provide a logical extension.

## Supporting Information

S1 Data Contains the TDC data as provided by the perfusion software used in this study.
(ZIP)
